# Pharmacokinetics and Tolerability of Intravenous Sulbactam-Durlobactam with Imipenem-Cilastatin in Hospitalized Adults with Complicated Urinary Tract Infections, Including Acute Pyelonephritis

**DOI:** 10.1128/AAC.01506-19

**Published:** 2020-02-21

**Authors:** Olexiy Sagan, Ruslan Yakubsevitch, Krassimir Yanev, Roman Fomkin, Emily Stone, Daniel Hines, John O’Donnell, Alita Miller, Robin Isaacs, Subasree Srinivasan

**Affiliations:** a Regional Clinical Hospital of Zaporizhzhia, Zaporizhzhia, Ukraine; b Grodno Regional Clinical Hospital, Hrodna, Belarus; c Clinic of Urology, Sofia, Bulgaria; d Saratov State Medical University, Saratov, Russia; e Spero Therapeutics, Cambridge, Massachusetts, USA; f Entasis Therapeutics, Inc., Waltham, Massachusetts, USA

**Keywords:** durlobactam, sulbactam, urinary tract infection, acute pyelonephritis

## Abstract

Durlobactam (DUR; ETX2514) is a novel β-lactamase inhibitor with broad-spectrum activity against Ambler class A, C, and D β-lactamases. Durlobactam restores the *in vitro* activity of sulbactam (SUL) against members of the Acinetobacter baumannii-A. calcoaceticus complex (ABC). Sulbactam (SUL)-durlobactam (SUL-DUR) is under development for the treatment of ABC infections. Eighty patients with complicated urinary tract infection (cUTI), including acute pyelonephritis (AP), were randomized 2:1 to receive SUL-DUR at 1 g/1 g intravenously (i.v.) or placebo every 6 h (q6h) for 7 days and background therapy with imipenem-cilastatin (IMI) at 500 mg i.v. q6h to evaluate the tolerability of SUL-DUR in hospitalized patients. Patients with bacteremia could receive up to 14 days of therapy. SUL-DUR tolerability and the values of various pharmacokinetic (PK) parameters were determined. Efficacy was recorded at the test-of-cure (TOC) visit. SUL-DUR was well tolerated, with no serious adverse events (AEs) being reported. Headache (5.7%), nausea (3.8%), diarrhea (3.8%), and vascular pain (3.8%) were the most common drug-related AEs with SUL-DUR and were mostly of mild or moderate severity. The PK profile of DUR and SUL in hospitalized patients was consistent with observations in healthy volunteers. Overall success in the microbiological modified intent-to-treat (m-MITT) population was similar between the groups, as would be expected with IMI background therapy in all patients (overall success at the TOC visit, 76.6% [*n* = 36] with SUL-DUR and 81.0% [*n* = 17] with placebo). SUL-DUR in combination with IMI was well tolerated in patients with cUTIs. The pharmacokinetics of SUL-DUR observed in hospitalized patients was similar to that observed in healthy volunteers. (This study has been registered at ClinicalTrials.gov under identifier NCT03445195.)

## Abstract (precis)

Durlobactam (DUR; ETX2514) is a novel β-lactamase inhibitor with broad-spectrum activity against Ambler class A, C, and D β-lactamases. Durlobactam restores the *in vitro* activity of sulbactam (SUL) against members of the Acinetobacter baumannii-A. calcoaceticus complex (ABC). Sulbactam (SUL)-durlobactam (SUL-DUR) is under development for the treatment of ABC infections.

## INTRODUCTION

Acinetobacter baumannii is identified by the Centers for Disease Control and Prevention and the World Health Organization to be a critical priority in need of new treatment options (1–3). A. baumannii belongs to a larger cluster of species that is referred to as the A. baumannii-A. calcoaceticus complex (ABC) and that has been associated with serious infections, including hospital-acquired bacterial pneumonia/ventilator-associated bacterial pneumonia (HABP/VABP), complicated urinary tract infections (cUTIs), bloodstream infections, and wound infections (4, 5). Approximately two-thirds of ABC infections are caused by multidrug-resistant (MDR) isolates (6–8). Serious infections caused by MDR ABC isolates are associated with high rates of morbidity (4, 9–12), and the rate of mortality may range up to 50% or higher (4, 13–15). Thus, an urgent need exists to identify new antimicrobial agents to treat serious ABC infections (8, 16).

Durlobactam (DUR; also known as ETX2514) is a novel, diazabicyclooctenone β-lactamase inhibitor (BLI) that exhibits potent inhibition of class A, C, and D β-lactamases (16–18). *In vitro*, durlobactam exhibits intrinsic antibacterial activity against some *Enterobacteriaceae* but has no significant intrinsic activity against ABC isolates. Sulbactam (SUL), a class A BLI, also exhibits antibacterial activity against ABC isolates; however, its use as monotherapy has been limited by increasing resistance (11). In preclinical studies, potent *in vitro* and *in vivo* activity against ABC isolates, including carbapenem-resistant ABC isolates, has been observed with sulbactam-durlobactam (SUL-DUR) (17, 19, 20), and this activity extends to isolates resistant to colistin (21, 22). SUL-DUR is being developed for the treatment of infections caused by ABC isolates, including MDR and carbapenem-resistant isolates.

Phase 1 clinical studies in healthy subjects evaluated the pharmacokinetic (PK) profiles of DUR after the administration of single and multiple ascending intravenous (i.v.) doses alone and in combination with SUL, the plasma and intrapulmonary concentrations of both components, and the drug-drug interaction potential between DUR and SUL (23–25). SUL-DUR is being developed for use in patients with Acinetobacter infections, the majority of whom are expected to be critically ill. Prior to dosing critically ill patients in a phase 3 study, assessment of the PK and tolerability of SUL-DUR in a phase 2 study with a hospitalized patient population was planned as a bridging strategy. This study evaluated the tolerability and PK of SUL-DUR in patients with cUTIs, including acute pyelonephritis (AP).

## RESULTS

Eighty patients were randomized to receive SUL-DUR (*n* = 53) or placebo (*n* = 27). Two patients in the SUL-DUR group discontinued the treatment due to an adverse event (AE). One patient had moderate urticaria on day 3 of administration of the study drug that was self-limiting. The second patient was an 82-year-old patient whose serum creatinine concentration increased from 69 μmol/liter (normal concentration, 55 to 127 μmol/liter) on day 1 to 138 μmol/liter on day 3 and 109 μmol/liter on day 5. The study drug was discontinued on day 7, as the protocol did not allow for adjustment of the dose of imipenem-cilastatin (IMI) or SUL-DUR for changes in creatinine clearance, which would have been necessary to safeguard this elderly patient.

At the baseline, the demographic and clinical characteristics of the patients in the treatment groups were generally comparable (Table 1). Two patients had bacteremia at the baseline.

**TABLE 1:** Baseline characteristics (ITT population)

Characteristic^*a*^	Value for the following treatment group:	
	SUL-DUR + IMI (*n* = 53)	Placebo + IMI (*n* = 27)
Mean ± SD age (yr)	51.4 ± 17.6	54.9 ± 15.9
No. (%) of female patients	27 (50.9)	11 (40.7)
No. (%) of white patients	53 (100.0)	27 (100.0)
No. (%) of Hispanic or Latino patients	1 (1.9)	0
Mean ± SD body wt (kg)	83.8 ± 20.6	85.8 ± 17.9
Mean ± SD body mass index (kg/m^2^)	28.1 (6.7)	28.6 (5.9)
Creatinine clearance (ml/min)	94.3 ± 23.8	91.7 ± 18.2
No. (%) of patients with:		
cUTI	31 (66.0)	16 (76.2)
Intermittent or indwelling catheter	3 (9.7)	2 (12.5)
Functional or anatomic abnormality	15 (48.4)	7 (43.8)
Complete or partial obstructive uropathy	15 (48.4)	4 (25.0)
Azotemia	3 (9.7)	0
Chronic urinary retention (men)	12 (38.7)	6 (37.5)
Acute pyelonephritis	16 (34.0)	5 (23.8)
No. (%) of patients with the following signs and symptoms:		
Fever with chills, rigors, or warmth	14 (87.5)	3 (60.0)
Nausea/vomiting within 24 h of screening	11 (68.8)	3 (60.0)
Dysuria, increased frequency, or urgency	10 (62.5)	5 (100.0)
Acute flank pain or costovertebral angle tenderness	15 (93.8)	5 (100.0)
No. (%) of patients with evidence of pyuria criteria		
Positive leukocyte esterase on urinalysis	9 (56.3)	2 (40.0)
WBC count of >10 cells/mm^3^ in unspun urine	6 (37.5)	0
WBC count of >10 cells/hpf in urine sediment	12 (75.0)	5 (100.0)

a WBC, leukocyte; hpf, high-power field.

### Tolerability.

The primary objective of this study was to assess the tolerability profile of SUL-DUR, which was generally well tolerated (Table 2). The numbers of patients experiencing at least 1 AE were 20 (37.7%) patients in the SUL-DUR group and 8 (29.6%) patients in the placebo group. The majority of AEs were mild or moderate in severity, with no serious AEs being reported. The most commonly reported drug-related AEs were headache, diarrhea, nausea, and phlebitis. The incidence of treatment-related AEs was similar between the treatment groups: 12 (22.6%) patients treated with SUL-DUR and 4 (14.8%) patients treated with placebo. One (1.9%) patient in the SUL-DUR group had severe nausea that was considered treatment related, but the patient continued in the study.

**TABLE 2:** Summary of adverse events (safety population)

Characteristic	No. (%) of patients in the following treatment group:	
	SUL-DUR + IMI (*n* = 53)	Placebo + IMI (*n* = 27)
Any AE	20 (37.7)	8 (29.6)
Any drug-related AE	12 (22.6)	4 (14.8)
Serious AEs	0	0
Deaths	0	0
Discontinuation for AE	2 (3.8)	0
Incidence of AEs		
Abdominal pain upper	1 (1.9)	1 (3.7)
Alanine aminotransferase concn increase	1 (1.9)	0
Blood creatinine concn increase	1 (1.9)	0
Blood glucose concn increase	1 (1.9)	0
Blood pressure increase	1 (1.9)	0
Bronchitis	1 (1.9)	0
Conjunctivitis	1 (1.9)	0
Diarrhea	2 (3.8)	0
Duodenitis	1 (1.9)	0
Dysbacteriosis	0	1 (3.7)
Dyspepsia	0	1 (3.7)
Gastritis	1 (1.9)	0
Glomerular filtration rate decrease	1 (1.9)	0
Headache	5 (9.4)	2 (7.4)
Infusion site reaction	1 (1.9)	0
Nausea	2 (3.8)	1 (3.7)
Oropharyngeal pain	1 (1.9)	1 (3.7)
Phlebitis	3 (5.7)	1 (3.7)
Pruritus	0	1 (3.7)
Pseudomembranous colitis	0	1 (3.7)
Respiratory tract infection, viral	1 (1.9)	0
Urticaria	1 (1.9)	0
Vascular pain	2 (3.8)	0
Vomiting	2 (3.8)	0
Vulvovaginal candidiasis	1 (1.9)	0

Two subjects discontinued treatment. A 35-year-old Caucasian female randomized to SUL-DUR experienced acute urticaria on study day 2. Treatment included prednisolone, diphenhydramine, and chloropyramine; the study medication was permanently discontinued on day 3, and the subject recovered from the event on day 5 but was discontinued from the study. The investigator considered the event to be moderate and to be related to the study drug. An 82-year-old Caucasian male randomized to SUL-DUR had central laboratory findings on day 1 of a serum creatinine concentration of 69 μmol/liter (normal concentration, 55 μmol/liter to 127 μmol/liter) and a creatinine clearance of 94 ml/min (normal creatinine clearance, >52 ml/min). There was a gradual increase in the creatinine concentration, and on day 7 the creatinine concentration was 107 μmol/liter and the creatinine clearance was 61 ml/min. The study medication was discontinued due to reduced creatinine clearance on day 7, as the protocol did not allow for imipenem dose reductions.

No clinically meaningful changes in safety laboratory data were noted. A larger change from the baseline in the mean leukocyte and neutrophil counts was observed in the SUL-DUR group than in the placebo group at the late follow-up (LFU) visit (−0.24 × 10^9^/liter versus −1.42 × 10^9^/liter for leukocytes and −4.96 × 10^9^/liter versus −8.22 × 10^9^/liter for neutrophils). However, the baseline leukocyte counts in these patients with infections were high, with the median in the SUL-DUR and placebo groups being 8.1 × 10^9^/liter versus 6.75 × 10^9^/liter. Moreover, no patients developed leukopenia or neutropenia. Mild changes in hepatic safety laboratory parameters from the baseline were observed at similar rates in patients in both groups. None of these changes were clinically significant or led to the discontinuation of therapy. No clinically meaningful changes in vital signs, electrocardiogram (ECG) findings, or physical findings were observed.

### Pharmacokinetics.

**FIG 1:**
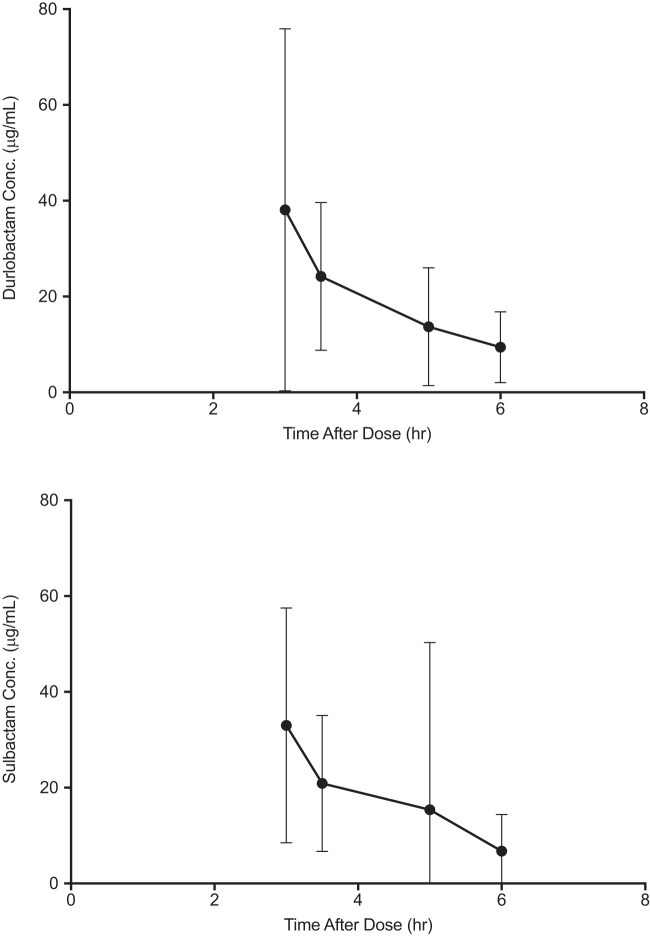
Mean (standard deviation) steady-state (day 4) plasma concentrations of durlobactam and sulbactam over a 6-h dosing interval.

The plasma concentrations of durlobactam and sulbactam in phase 2 study subjects were comparable over the 6-h sampling interval (Fig. 1). The values of the PK parameters of durlobactam and sulbactam at steady state were generally consistent when administered as a 1:1 ratio of 1,000 mg plus 1,000 mg infused over 3 h every 6 h (Table 3). Mean elimination half-lives of 2.2 and 1.6 h for durlobactam and sulbactam, respectively, resulted in accumulation indexes of 1.2 and 1.1, respectively. The mean steady-state clearance (CL_ss_) and volume of distribution (*V*_ss_) of durlobactam were 10.3 liters/h and 31.6 liters, respectively. These values were similar to the mean clearance and volume of distribution estimates for sulbactam (13.4 liters/h and 36.0 liters, respectively). The variability of the PK parameter estimates for clearance and volume of distribution were higher for sulbactam (62.3% and 64.9%, respectively) than for durlobactam (38.9% and 41.6%, respectively).

**TABLE 3:** Mean values of PK parameters for durlobactam and sulbactam following a 3-h i.v. infusion of 1,000 mg each^*a*^

Parameter	Durlobactam		Sulbactam	
	Mean ± SD	% CV	Mean ± SD	% CV
*k*_el_ (liter/h)	0.43 ± 0.25	58.0	0.54 ± 0.28	51.3
Half-life (h)	2.2 ± 1.6	72.9	1.6 ± 1.1	66.0
*T*_max_ (h)	3.1 ± 0.5	16.2	3.2 ± 0.6	17.9
*C*_max_ (μg/ml)	39.9 ± 38.2	95.8	39.1 ± 38.6	98.7
*C*_min_ (μg/ml)	8.9 ± 6.7	74.7	6.5 ± 7.2	111.9
CL_ss_ (liters/h)	10.6 ± 4.0	38.9	13.4 ± 8.4	62.3
*V*_ss_ (liters)	31.6 ± 13.1	41.6	36.0 ± 23.4	64.9
AUC_0–tau_ (μg·h/ml)	123.8 ± 85.7	69.2	107.8 ± 83.1	77.1
Accumulation index	1.2 ± 0.3	27.4	1.1 ± 0.2	18.3

a Data represent the mean ± standard deviation (*n* = 45 for *k*_el_, half-life, and the accumulation index; *n* = 52 for all other parameters). CV, coefficient of variation; *k*_el_, first-order elimination rate constant associated with terminal (log-linear) portion of the curve; *T*_max_, time after dosing at which the maximum concentration was observed; *C*_max_, maximum observed concentration measured after dosing; *C*_min_, minimum observed concentration measured after dosing; CL_ss_, steady-state clearance; *V*_ss_, steady-state volume of distribution; AUC_0–_τ, steady-state area under the concentration-versus-time curve from the dosing time to the dosing time plus τ, using the linear up-log down method; accumulation index =$1/\left(1-e^{-k_{\text{el}}\cdot \tau }\right)$.

### Efficacy.

The overall rates of success in the microbiological modified intent-to-treat (m-MITT) population were similar in both groups, 36 (76.6%) patients in the SUL-DUR group and 17 (81.0%) patients in the placebo group, as would be expected with a background therapy with IMI in all patients. Overall success in the microbiologically evaluable (ME) population occurred in 36 (80.0%) patients in the SUL-DUR group and 17 (81.0%) patients in the placebo group.

Seven patients presented with a baseline IMI-nonsusceptible (IMI-NS; defined as an MIC of ≥2 mg/liter) Gram-negative pathogen. For these patients, the overall success at the test-of-cure (TOC) visit occurred in three of three (100%) patients in the SUL-DUR group (one infected with an IMI-NS Proteus mirabilis isolate and two infected with IMI-NS Pseudomonas aeruginosa isolates) and in three of four (75%) patients in the placebo group (two infected with IMI-NS P. aeruginosa isolates and one of two [50%] infected with IMI-NS Klebsiella pneumoniae isolates).

## DISCUSSION

This was the first study of SUL-DUR in hospitalized patients in which all patients received background therapy with IMI, in addition to SUL-DUR or placebo. SUL-DUR was generally well tolerated and had a tolerability profile similar to what had been observed in healthy volunteers. Previous studies have shown that DUR with and without SUL and IMI is well tolerated in healthy volunteers (23). The majority of AEs in the SUL-DUR group were mild or moderate in severity, with no serious AEs being reported. As would be expected with a background of therapy with IMI, the clinical and microbiological outcomes were comparable between the treatment groups. Additionally, the values of the PK parameters in this population were similar to those observed in healthy volunteers (23–25). A population pharmacokinetic (PPK) analysis and pharmacokinetic-pharmacodynamic target attainment (PK-PD TA) analysis have recently been conducted for SUL-DUR in preparation for phase 3 trial dose justification (26). Plasma concentration data from a phase 1 single- and multiple-ascending-dose study and a phase 1 renal impairment study were utilized in the construction of a DUR PPK model. For SUL, a published PPK model was utilized in support of the dose justification. For both DUR and SUL, visual predictive checks (VPCs) of model-based simulations against the observed phase 2 trial data presented here were used as a qualification of the base structural models and the PK-PD TA analyses. Both PPK models were found to predict the observed phase 2 trial plasma SUL-DUR concentration data quite well, with an excellent probability of PK-PD TA of >90% against pathogens with a SUL-DUR MIC of ≤4 mg/liter.

SUL-DUR is being developed for patients with serious infections due to members of the ABC, including HABP/VABP. For infections caused by ABC isolates, high rates of multidrug resistance contribute to high morbidity, extended hospitalization, and excess mortality (4, 6, 27). Currently, colistin is the only antibiotic with consistent activity against ABC strains. Mortality rates among patients with HABP/VABP treated with colistin-based regimens remain high at about 40% (28), and the doses are limited by toxicity issues. A critical unmet need for novel and safer approaches to the treatment of infections caused by ABC isolates remains.

Limitations of this study include the inability to comment on the efficacy of SUL-DUR. This agent is a narrow-spectrum antimicrobial designed to treat highly resistant strains of ABC causing infections; however, as expected, no patient with an ABC infection was enrolled in this study. DUR is a broad-spectrum BLI and has been shown to restore the activity of IMI against carbapenem-resistant Gram-negative isolates. A *post hoc* sequencing analysis and susceptibility testing of the small number of IMI-NS isolates in this study revealed that the genomes of six of the seven IMI-NS isolates encoded one or more carbapenemase genes, and addition of DUR *in vitro* restored IMI susceptibility to all six. While these results are indirect, they support the hypothesis that β-lactamase inhibition by DUR can be clinically effective among carbapenem-resistant Gram-negative pathogens.

In conclusion, SUL-DUR was generally well tolerated in moderately ill, hospitalized adults with cUTIs or AP when administered with a background therapy of IMI. Based on the PK established in the phase 2 trial described here and in support of PPK models, PK-PD TA analyses suggest that optimal target attainment against members of the ABC is achieved with a SUL-DUR dose of 1,000 mg (of each component) administered every 6 h (q6h) via a 3-h i.v. infusion (26). This dose is currently being studied in a global phase 3 study of efficacy and safety for treating serious infections due to ABC isolates in hospitalized patients.

## MATERIALS AND METHODS

Patients were enrolled at 20 clinical sites in Belarus, Bulgaria, Russia, and Ukraine between January 2018 and May 2018. The study was conducted in accordance with the Declaration of Helsinki and Good Clinical Practices. The study protocol and amendments were approved by an institutional review board for each clinical site, and all patients provided written informed consent prior to any study procedure. This study was registered at ClinicalTrials.gov under identifier NCT03445195.

### Study design.

This was a double-blind, randomized, placebo-controlled study to evaluate the tolerability and PK of i.v. SUL-DUR administered with IMI in patients with cUTIs, including AP, who were otherwise relatively healthy.

### Study treatments.

Patients were randomized 2:1 to receive either SUL-DUR at 1 g/1 g i.v. or matching placebo. The treatments were reconstituted and diluted in 100 ml of 0.9% saline and then infused over 3 h every 6 h (q6h) for 7 days (28 doses). All patients received background therapy with IMI at 500 mg i.v. infused over 30 min q6h. Patients with bacteremia could receive up to 14 days of therapy. Randomization was stratified by baseline diagnosis (symptomatic cUTI versus AP), and at least 30% of the patients were required to have a diagnosis of AP at study entry.

### Patient selection.

Male or female patients ages 18 to 90 years who were expected to require hospitalization and treatment with i.v. antibiotics for cUTI were eligible. A documented or suspected cUTI was defined based on the presence of at least two signs and symptoms, a urine specimen with evidence of pyuria, and the presence of at least one risk factor. Documented or suspected AP was defined by at least two signs or symptoms and a urine specimen with evidence of pyuria. Women of childbearing potential were required to have a negative pregnancy test before randomization and to use 2 highly effective methods of contraception until at least 30 days after the last dose of study drug. Men were required to use adequate contraception for at least 90 days after the last dose of study drug. Patients were excluded for the presence of any disease or condition that could confound the assessment of efficacy, including the use of any systemic antibiotic active against Gram-negative uropathogens for more than 24 h in the 72-h period prior to randomization.

### Study assessments.

Tolerability was assessed from the incidence of treatment-emergent adverse events (TEAEs) and evaluation of changes from the baseline in the findings of clinical laboratory tests (serum chemistry, hematology, urinalysis), 12-lead electrocardiogram (ECG), vital signs (heart rate, blood pressure, respiratory rate), and physical examination, including weight.

Sparse sampling for PK analysis was completed, with samples being obtained predose on day 1, postdose of study drug on day 4 (±1 day) at the end of the infusion, and 0.5, 2, and 3 h after the end of the infusion (prior to the start of the next infusion). PK concentrations were analyzed using a validated liquid chromatography-tandem mass spectrometry assay (data on file, Covance Laboratories, Inc.).

Clinical signs and symptoms were assessed at screening, on days 2 through 6, at the end of treatment (EOT; 7 to 14 days after completing treatment), at the TOC visit (7 days post-EOT), and at the late follow-up (LFU) visit (7 days post-TOC visit).

### Statistical analysis.

No formal sample size calculation was performed. At least 80 patients were expected to be randomized 2:1 to SUL-DUR or placebo. The intent-to-treat (ITT) population included all randomized patients. The modified intent-to-treat (MITT) population included patients who met the criteria for the ITT population and received any study drug and was used as the population for the primary analysis of tolerability.

The values of the pharmacokinetic parameters in the PK population (the MITT population with at least one plasma PK sample drawn) were estimated by noncompartmental analysis, which was completed using Phoenix PK software (WinNonlin), version 8.1. The area under the plasma DUR or SUL concentration-versus-time curves (AUC) was calculated using the linear up-log down method. Where the first-order elimination rate constant (*k*_el_) could not be estimated, half-life, steady-state volume of distribution (*V*_ss_), and accumulation index were not reported. The PK parameters evaluated included half-life, the time after dosing at which the maximum concentration was observed (*T*_max_), the maximum observed concentration measured after dosing (*C*_max_), the minimum observed concentration measured after dosing (*C*_min_), *k*_el_, *V*_ss_, the steady-state clearance (CL_ss_), the steady-state area under the concentration-versus-time curve from the dosing time to the dosing time plus τ (AUC_0–τ_) (where τ is the dosing interval), and the accumulation index. The PK parameters were reported using descriptive statistics.
